# Supplementary material for: Reduction in the Dietary VA Status Prevents Type 2 Diabetes and Obesity in Zucker Diabetic Fatty Rats
Source: Biomolecules. 2022 Mar 31;12(4):528. doi: 10.3390/biom12040528 (PMC9032907; doi:10.3390/biom12040528)
Supplement: Supplementary file 1 [file biomolecules-12-00528-s001.zip › biomolecules-1620239-supplementary.pdf]

Supplemental materials

Table S1: Real-time PCR primer sequences of the indicated genes

Gene name	Forward Sequence	Reverse Sequence
Gck	5'-CCTGGGCTTCACCTTCTCCTT-3'	5'-GAGGCCTTGAAGCCCTTGGT-3'
Srebp-1c	5′-GGAGCCATGGATTGCACATT-3′	5′-AGGCCAGGGAAGTCACTGTCT-3′
Srebp 2	5'-CTGCAGATCCCGCAGTACAG-3'	5'-GGTGGATGAGGGAGAGAAGGT-3'
Pck1	5'-AGTCACCATCACTTCCTGGAAGA-3'	5'-GGTGCAGAATCGCGAGTTG-3'
Fas	5'-CAGGAACTGAACGGCATTACCTC-3'	5'-CATTTTCTAGGGATAACAGCAC-3'
Acl	5'-GCTGAAGACATTAAGAGACACCTGTT-3'	5'-AAATTGAATAGGCCGGAGATGA-3'

Gck, glucokinase; *Srebp-1c*, Sterol regulatory element-binding protein 1; *(Srebp2),* Sterol regulatory element-binding protein 2; *(Pck1),* Phosphoenolpyruvate Carboxykinase 1; *Fas,* Fatty acid synthase; *Acl,* ATP citrate lyase.

Table S2: Antibody sources and dilution folds

**Protein name**	**Catalog No.**	**Dilution fold**	**Manufacture**
Fatty acid synthase (FAS)	#3180S	1: 1000	Cell Signaling Technology
ATP citrate lyase (ACL)	#4332S	1: 1000	Cell Signaling Technology
Glucokinase (GCK)	#sc-7908	1: 500	Santa Cruz Biotechnology Inc
Phosphoenolpyruvate carboxykinase (PEPCK-C)	#10004943	1: 1000	Cayman Chemical
Insulin receptor β subunit (IRβ)	#610109	1: 1000	BD Biosciences

β-actin	#4970 s	1: 1000	Cell Signaling Technology
Anti-rabbit IgG, HRP-linked Antibody	#7074	1: 1000	

Note: Antibodies are diluted in TBST (10 mM Tris, 150 mM NaCl, 0.1% Tween 20, pH 7.5) containing 5% bovine serum albumin or 5% non-fat milk. All antibodies were used according to the manufacturer's instructions.
